# Study of Monocyte-High Density Lipoprotein ratio to coronary artery diameter in coronary heart disease patients

**DOI:** 10.21542/gcsp.2025.11

**Published:** 2025-02-28

**Authors:** Azhari Gani, Muhammad Diah Yusuf, Siti Adewiyah, Malahayati

**Affiliations:** 1 Department of Internal Medicine, Faculty of Medicine, Universitas Syiah Kuala and Dr. Zainal Abidin Hospital, Banda Aceh, Indonesia; 2 Internal Medicine Specialist Education Program, Faculty of Medicine, Universitas Syiah Kuala and Dr. Zainal Abidin Hospital, Banda Aceh, Indonesia

## Abstract

Background: The Monocyte to High-Density Lipoprotein Ratio (MHR) ratio reflects the proatherogenic and antiatherogenic balance and increased MHR values are related to coronary atherosclerosis obstruction’s presence, progressivity, and severity. This study uses medical records and coronary angiography data to assess the correlation between MHR and coronary artery diameter in coronary heart disease patients.

Methods: This study involved data from 230 patient medical records. The data collection approach was a cross-section design, and total sampling was performed by citing medical records of patients who underwent coronary angiography at the catheterization installation of Dr. Zainal Abidin Hospital, Banda Aceh, Indonesia.

Results: The multivariate test showed that MHR and the coronary slow flow group, MINOCA, and stenosis were significant with a value of *p* < 0.0001. The results of the bivariate test showed the relationship between MHR and coronary artery diameter in all three groups. Coronary slow flow, MINOCA, and stenosis were insignificant *p* > 0.05.

Conclusion: This study shows that MHR can be a suitable, inexpensive, fast parameter for identifying the severity of coronary atherosclerosis obstruction.

## Introduction

Coronary heart disease (CHD) is the leading cause of death worldwide, with a WHO report stating that 17.9 million people died in 2016 from cardiovascular disease, with heart attacks and strokes accounting for 85% of these deaths^1^. In Indonesia, the prevalence of CHD increased from 0.5% in 2013 to 1.5% in 2018, with Aceh having a prevalence of 1.6%.

Coronary heart disease occurs due to the narrowing of the coronary arteries by atherosclerosis, which reduces perfusion to the myocardium^2^. This process involves inflammation, oxidative stress, platelet activation, and endothelial dysfunction^3^. Monocytes and macrophages play an essential role in atherosclerosis, where activated monocytes convert oxidized LDL into foam cells, contributing to atherosclerosis progression^4^. In contrast, HDL is protective by reducing monocyte activation and inflammation^5^.

Inflammation, oxidative stress, platelet activation, and endothelial dysfunction are the underlying processes of atherosclerosis progression^6^. Changes in the number and composition of white blood cells are often associated with inflammatory conditions that can be used as predictive and prognostic factors in cardiovascular patients, with monocytes being the subset of white blood cells with which the association is strongest^7^. The high value of monocyte count during the acute phase of acute myocardial infarction is related to the progression of plaque itself^8^. Monocytosis has been assessed as an independent marker for coronary heart disease and acute myocardial infarction^9^.

Monocyte to High-Density Lipoprotein Ratio (MHR) has been identified as a novel marker effective in predicting and evaluating cardiovascular risk^10^. MHR is more economical than other inflammatory markers and is an independent predictor of cardiovascular events in a variety of conditions, including myocardial infarction and acute kidney disease^11^. The MHR test shows high sensitivity and specificity and is less expensive than the hs-CRP test. Studies in various countries, including Indonesia, show that high MHR is associated with more severe atherosclerosis and the risk of coronary heart disease^12^. This study assesses the relationship between increased monocytes and decreased HDL levels indicated by high MHR values as a risk factor in CHD patients.

## Methodology

### Research design

This study is an analytical observational study that uses a cross-sectional study *approach* to assess the correlation of MHR in the degree of coronary artery diameter measurement carried out in 2020–2023 at the Internal Medicine Cardiology Division of dr. Zainoel Abidin Banda Aceh Hospital.

### Research sample

**Figure 1.**
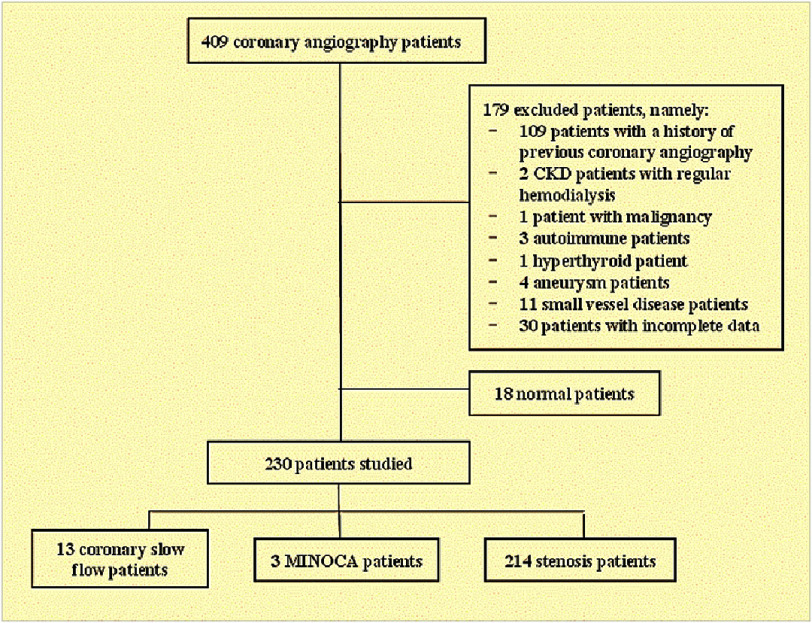
Selection of research subjects.

The study sample consisted of all patients who underwent coronary angiography at the Catheterization Installation of Dr. Zainoel Abidin Banda Aceh Hospital and met the relevant inclusion and exclusion criteria (Figure 1). The inclusion criteria were patients diagnosed with CHD who had undergone coronary angiography, Age over 18 years, and availability of laboratory data (routine blood tests, lipid profile). The exclusion criteria were patients who had previously undergone coronary angiography, patients with malignancy, liver disorders, thyroid dysfunction, autoimmune diseases, kidney dysfunction requiring hemodialysis, and those suffering from sepsis. Patients with coronary artery aneurysms, small vessel disease in the coronary artery, and those with incomplete data were excluded.

### Research procedure

**Figure 2.**
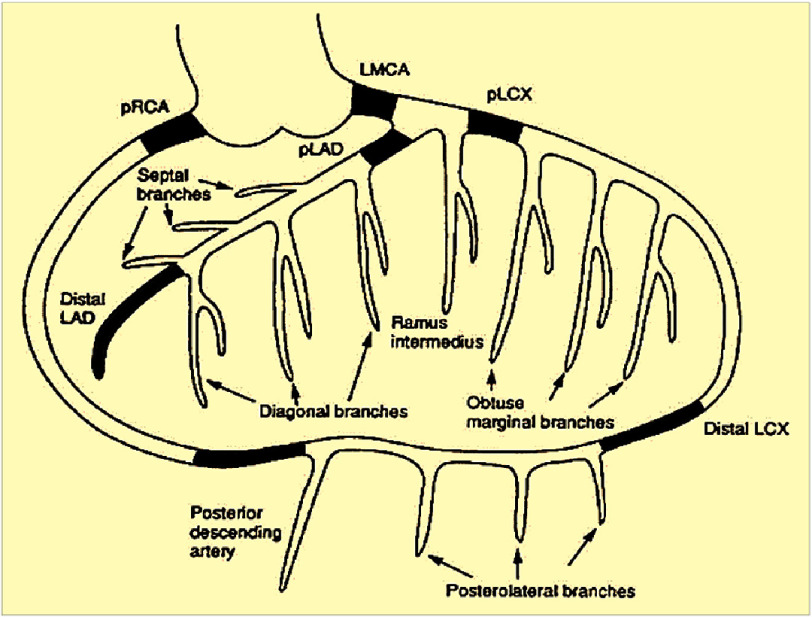
Coronary artery measurement location. Left main on the Proximal Distal part of the LAD place (Left Anterior Descending), LCX (Left Circumflex), and RCA (Right Coronary Artery).

The research implementation began with a survey of medical record data of CHD patients who underwent coronary angiography and had complete medical records in the medical record room and catheterization installation of Dr. Zainoel Abidin Banda Aceh Hospital. Data collection was carried out by citing medical records. The data collected included name, Age, gender, comorbid diseases, and laboratory results such as routine blood tests and lipid profiles. Coronary artery measurements were performed using the Stenosis Analysis application on a GE Innova 3131 coronary angiography machine^13^. The location of the coronary artery measurement is shown in Figure 2.

### Statistical analyses

Data analysis using SPSS statistical software and univariate analysis describes the frequency of each dependent and independent variable as a frequency distribution table. The bivariate analysis looked for a relationship between independent and dependent variables using One Way ANOVA (Parametric) and Kruskal Wallis (Non-Parametric) with a p of <0.05 considered significant. The analysis of the relationship between the research variables using Pearson Correlation with *r* = 1 is a strong relationship. Confirmation for each elementary school group with different historical backgrounds, Bootstrap analysis was carried out.

### Ethics approval

We obtained written approval from the ethics committee of Dr Zainal Abidin Hospital with ethics approval: 068/EA/FK-RSUDZA/2022.

## Results

A total of 230 CHD patients who met the inclusion and exclusion criteria and underwent coronary angiography at Dr. Zainoel Abidin Hospital were evaluated in this study. The patients were divided into three groups based on their coronary angiography results: coronary slow flow, myocardial infarction with nonobstructive coronary arteries (MINOCA), and stenosis. The basic characteristics of the patients are shown in Table 1, which depicts the demographic and comorbid attributes of the three groups: coronary slow flow, MINOCA, and stenosis. Among the 13 patients with coronary slow flow, the majority were men (84.6%), with most being in the age group of 50–59 years (76.9%) and a median age of 55.0 ± 4.3 years. The majority of these patients had hypertension (76.9%) and a median BMI of 21.4 ± 2.6 kg/m^2^. The MINOCA group consisted of 3 patients. All were men aged 50–59 years, with a median age of 54.0 ± 4.2 years. Only one patient in this group had hypertension, and all were in the BMI range of 18.5–24.9 kg/m^2^ with a median BMI of 20.3 ± 0.4 kg/m^2^. The stenosis group included 214 patients, mostly men (86.9%). The age distribution varied, but most were in the 50–59 age group (48.5%) with a median age of 54.0 ± 9.2 years. This group had a prevalence of hypertension (41.1%) and diabetes mellitus (35.5%), with most patients having a BMI in the range of 18.5–24.9 kg/m^2^ (66.8%) and a median BMI of 22.8 ± 4.4 kg/m^2^.

**Table 1:** Basic characteristics of the research sample.

Variable	Coronary slow flow n (%)	MINOCA n (%)	Stenosis n (%)
Gender			
Man Woman	11 (84,6) 2 (15,4)	3 (100) 0 (0)	186 (86,9) 28 (13,1)
Age (years)			
<30 30–39 40–49 50–59 60–69 ≥ 70 Mean ± SD	0 (0) 0 (0) 0 (0) 10 (76,9) 3 (23,1) 0 (0) 55.0 ± 4.3	0 (0) 0 (0) 0 (0) 3 (100) 0 (0) 0 (0) 54.0 ± 4.2	2 (0,9) 10 (4,7) 44 (20,6) 104 (48,5) 44 (20,6) 10 (4,7) 54.0 ± 9.2
Comorbidities			
Hypertension Diabetes Mellitus	10 (76,9) 1 (7,7)	1 (33,3) 0 (0)	88 (41,1) 76 (35,5)
IMT (kg/m^2^)			
<18.5 18,5–24,9 ≥25 Mean ± SD	2 (15,4) 10 (76,9) 1 (7,7) 21.4 ± 2.6	0 (0) 3 (100) 0 (0) 20.3 ± 0.4	14 (6,6) 143 (66,8) 57 (26,6) 22.8 ± 4.4

Table 2 presents the bootstrap analysis results for various clinical and laboratory parameters across three patient groups: stenosis, coronary slow flow, and MINOCA. The stenosis group shows higher mean values for leukocytes, LDL, and triglycerides, while the MINOCA group has the highest total cholesterol and monocyte-HDL ratio (RMH). The coronary slow flow group exhibits higher HDL levels and larger coronary artery diameters (LAD distal) than the other groups. The 95% confidence intervals (CI) provide stable estimates for each parameter, with the stenosis group displaying more consistent results due to its larger sample size. This table highlights the distribution differences among groups, supporting further analysis of the risk and characteristics of coronary heart disease.

**Table 2:** Bootstrap analysis for three CHD groups with 95% CI.

Parameter	Stenosis (Mean ± CI)	Coronary Slow Flow (Mean ± CI)	MINOCA (Mean ± CI)
Leukosit (x10^3^ sel/mm^3^)	9800 (9700–9900)	8800 (8700–8900)	8000 (7800–8200)
Relative Monocytes (%)	9 (8.9–9.1)	7 (6.8–7.2)	10 (9.8–10.2)
Monosit Absolut (sel/mm^3^)	783 (780–786)	678 (672–684)	800 (796–804)
Kolesterol Total (mg/dl)	174 (172–176)	147 (145–149)	195 (194–196)
LDL (mg/dl)	115 (113–117)	85 (83–87)	130 (129–131)
HDL (mg/dl)	37 (36–38)	40 (39–41)	33 (32.5–33.5)
Triglycerides (mg/dl)	140 (138–142)	99 (97–101)	150 (148–152)
Rasio Monosit-HDL (RMH)	24.0 (23.8–24.2)	16.3 (15.7–17.0)	24.1 (23.5–24.7)
LAD Diameter (mm, Distal)	2.0 (1.9–2.1)	3.0 (2.9–3.1)	3.0 (2.8–3.2)
RCA Diameter (mm, Proximal)	3.0 (2.9–3.1)	3.0 (2.9–3.1)	3.0 (2.8–3.2)
BMI (kg/m^2^)	22.8 (22.5–23.1)	21.4 (20.9–21.9)	20.3 (20.0–20.6)

**Table 3:** Values of inflammatory cells and blood cholesterol in the medical records of research subjects.

**Variable**	Coronary slow flow (*n* = 13)	MINOCA (*n* = 3)	Stenosis (*n* = 214)	^*^p-**value**
	(Mean ± SD)	(Mean ± SD)	(Mean ± SD)	
Leukosit (x10^3^ sel/mm^3^)	8.800 ± 2.425	8.000 ± 1.311	9.800 ± 7.936	0,15
Relative Monocytes (%)	7 ± 3.2	10 ± 2	9 ± 2.4	0,36
Monosite absolute (sel/mm^3^)	678 ± 215.7	800 ± 36.1	783 ± 734.1	0,18
Kolesterol Total (mg/dl)	147 ± 45.1	195 ± 4.6	174 ± 114.6	0,23
LDL (mg/dl)	85 ± 52.2	130 ± 10.9	115 ± 40.8	0,17
HDL (mg/dl)	40 ± 17.1	33 ± 0.6	37 ± 12.1	0,31
Triglycerides	99 ± 44.7	150 ± 13.0	140 ± 76.5	0,06

**Notes.**

* One Way Anova.

**Table 4:** Comparison of coronary diameter in the three groups of respondents.

**Variable (mm)**	Coronary slow flow (*n* = 13)	MINOCA (*n* = 3)	Stenosis (*n* = 214)	^*^p-**value**
	(Mean ± SD)	(Mean ± SD)	(Mean ± SD)	
LM	4.0 ± 0.52	3.0 ± 0.58	3.5 ± 0.52	0.0008
Proximal LAD	3.5 ± 0.45	3.0 ± 0.29	3.0 ± 0.80	<0.0001
LAD distal	3.0 ± 0.26	3.0 ± 0.14	2.0 ± 1.07	<0.0001
LCx proximal	3.5 ± 0.31	3.0 ± 0.0	3.0 ± 0.47	<0.0001
LCx distal	2.75 ± 0.18	2.5 ± 0.0	2.0 ± 0.75	<0.0001
Proximal RCA	3.5 ± 0.38	2.5 ± 0.76	3.0 ± 0.49	0.0004
RCA distal	3.0 ± 0.19	2.0 ± 0.76	2.5 ± 1.03	0.0122

**Notes.**

* Kruskal Wallis Test

Table 3 shows the values of inflammatory cells and blood cholesterol of the study subjects in three groups of patients: coronary slow flow, MINOCA, and stenosis. The mean number of leukocytes and monocytes, both relative and absolute, was higher in MINOCA and stenosis patients compared to coronary slow-flow patients. However, these differences were not statistically significant. Mean levels of total cholesterol, LDL, and triglycerides were also higher in MINOCA and stenosis patients, but there was no significant difference between the three groups. HDL levels were low in all groups of patients. Overall, no parameters showed a statistically significant difference with a p-value of >0.05.

Table 4 shows significant differences in coronary artery diameter between three groups of patients: coronary slow flow, MINOCA, and stenosis. The coronary slow flow group had a larger average diameter in all vascular segments than the MINOCA and stenosis groups. The diameters of LM, proximal and distal LAD, proximal and distal LCx, and proximal and distal RCA in the MINOCA and stenosis group were significantly smaller with a p-value < 0.05, indicating that patients with coronary slow flow had wider coronary vessels than patients with MINOCA and stenosis.

Table 5 shows a significant difference in coronary artery diameter between stenosis patients with comorbidities of diabetes mellitus (DM) and hypertension. Patients with DM had smaller coronary artery diameters compared to patients with hypertension in several vascular segments, including proximal LAD (p=0.020), distal LAD (p<0.0001), proximal LCx (p=0.0316), distal LCx (p=0.011), proximal RCA (p=0.008), and distal RCA (p<0.0001). The diameter of LM showed no significant difference (p=0.129). These results suggest that patients with DM have a higher risk for narrowing of the coronary arteries than patients with hypertension.

**Table 5:** Coronary diameter in the stenosis group with comorbidities of diabetes mellitus and hypertension.

**Variable (mm)**	Stenosis Group (*n* = 214)		***p***-**value**
	Diabetes Mellitus (Median ± SD)	Hypertension (Median ± SD)	
LM	3.5 ± 0.56	3.5 ± 0.53	0,129
Proximal LAD	3.0 ± 0.40	3.0 ± 0.57	0,020
LAD distal	2.0 ± 0.34	2.5 ± 1.19	<0,0001
LCx proximal	3.0 ± 0.40	3.0 ± 0.41	0,0316
LCx distal	2.0 ± 0.72	2.5 ± 0.48	0,011
Proximal RCA	3.0 ± 0.47	3.0 ± 0.41	0,008
RCA distal	2.0 ± 0.50	2.75 ± 0.47	<0,0001

**Notes.**

* Mann Whitney Test

**Figure 3.**
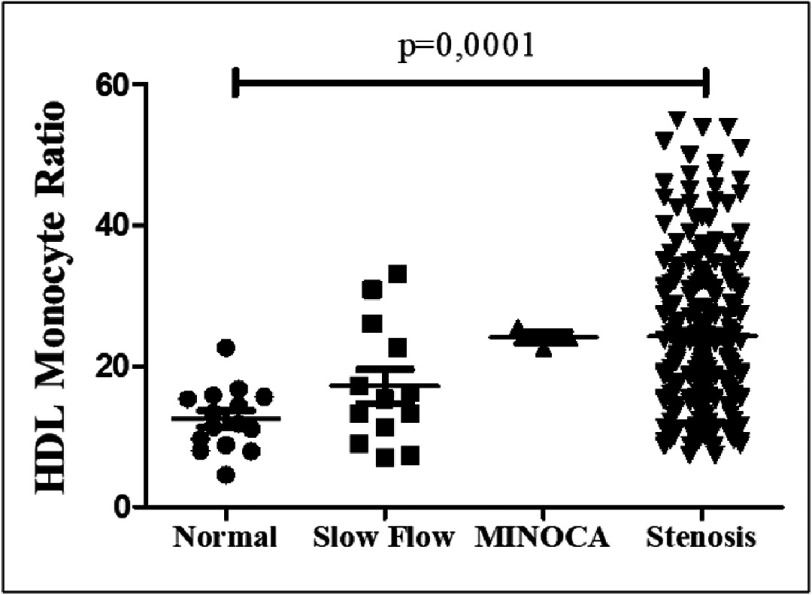
Dot-plot graph comparing MHR values in patient groups. Each dot represents each patient being assessed.

Figure 3 is a dot-plot graph comparing HDL Monocyte Ratio (MHR) values in patients with standard coronary angiography, slow flow coronary (slow flow), MINOCA, and stenosis. Each dot represents a patient. The one-way ANOVA analysis showed that the MHR value increased significantly in the group with coronary obstruction (slow flow, MINOCA, and stenosis) compared to the regular group, with *p* = 0.0001. Patients with stenosis had the highest MHR values, followed by the slow flow and MINOCA groups. These results suggest that higher MHR values are associated with increased coronary obstruction in CHD patients, and this difference is statistically significant.

Table 5 presents the Pearson correlation results between the Monocyte-HDL Ratio (MHR) and coronary artery diameter across three CHD patient groups: coronary slow flow, MINOCA, and stenosis. In the coronary slow flow group, the correlation between MHR and all coronary artery segments was weak and statistically insignificant (r ranging from −0.172 to 0.377, *p* > 0.05). In the MINOCA group, a strong negative correlation was observed between MHR and the diameter of the LM and proximal LAD (*r* = −0.894), but the results were not statistically significant (*p* > 0.05), likely due to the very small sample size. In the stenosis group, the correlation between MHR and all coronary artery segments was also weak and not significant (*r* ranging from −0.001 to 0.102, *p* > 0.05). Overall, the table indicates no significant relationship between MHR and coronary artery diameter in any group, suggesting that other factors, such as vascular remodeling or local plaque characteristics, may play a more dominant role in influencing coronary artery diameter in CHD patients.

**Table 6:** Correlation of Pearson ratio-HDL monocytes with coronary artery diameter.

**Variable**	Pearson Correlation					
	Coronary slow flow (*n* = 13)		MINOCA (*n* = 3)		Stenosis (*n* = 214)	
	r	*p-value*	r	*p-value*	r	*p-value*
MHR vs LM	−0,172	0,574	−0,894	0,295	0,099	0,150
MHR vs proximal LAD	0,022	0,943	−0,894	0,295	0,020	0,764
MHR vs LAD distal	0,338	0,259	−0,835	0,371	0,102	0,138
MHR vs LCx proximal	−0,149	0,627	0.210	0.327	0,053	0,440
MHR vs LCx distal	−0,014	0,963	−0.012	0.721	−0,001	0,988
MHR vs Proximity RCA	0,377	0,204	−0,992	0,083	−0,020	0,768
MHR vs RCA distal	0,377	0,432	−0,992	0,083	0,003	0,964

The relationship between the Monocyte-HDL Ratio (MHR) and various coronary artery segment diameters (LM, LAD proximal and distal, LCx proximal and distal, RCA proximal and distal) in three CHD patient groups (stenosis, coronary slow flow, and MINOCA) provides valuable insights into the association of systemic inflammation with structural vascular changes (Table 6). The LM segment, as the main artery supplying the LAD and LCx, is crucial hemodynamically, and any reduction in its diameter due to inflammation or atherosclerosis can increase the risk of global ischemia. Similarly, the LAD proximal, a segment with high susceptibility to atherosclerosis, is critical for CHD prognosis, as a smaller diameter is linked to a higher risk of myocardial infarction, particularly in stenosis and MINOCA patients. The LAD distal, with its smaller diameter, may be more vulnerable to systemic inflammation, potentially contributing to microvascular ischemia. The LCx proximal segment, supplying the lateral left ventricle, and LCx distal, with a smaller diameter, reflect the impact of systemic inflammation on perfusion, especially in slow coronary flow and mild stenosis cases. The RCA proximal, responsible for perfusion of the right heart and posterior wall, and the RCA distal, prone to inflammation-induced narrowing, are key areas for evaluating ischemic risk in distal distributions.

Clinically, these correlations highlight MHR as a potential predictor of coronary segment diameter changes. Significant relationships in specific segments, such as LM or LAD proximal, may pinpoint areas most affected by systemic inflammation. This understanding can prioritize angiographic evaluations and therapeutic strategies for CHD patients with elevated MHR, offering a segmental approach to assessing the impact of inflammation and lipid dysregulation on vascular structure.

## Discussion

This study uses a cross-sectional design with a total sampling method for samples that meet the inclusion and exclusion criteria. The parameters measured included coronary angiography medical record data, routine blood tests, lipid profiles, and coronary artery diameter, aiming to evaluate the relationship between the monocyte-HDL (MHR) ratio and coronary artery diameter. An increase in MHR has been associated with the progression and progression of atherosclerosis and may be a marker for identifying patients at high risk of major cardiovascular events. Previous research has shown a positive association between MHR and the severity of coronary atherosclerosis^14^.

The 230 study samples, there were 13 patients in the coronary slow flow group, 3 in the MINOCA group, and 214 in the stenosis group (Table 1). The number of males was more dominant in all groups, consistent with the study of Hung et al. (2023), which showed that women had a lower prevalence and severity of coronary artery calcification^15^. The risk of CHD in women increases after menopause due to a decrease in the protective effects of estrogen. The age group of 50–59 years had the highest number of patients: 10 patients (76.9%) in coronary slow flow, three patients (100%) in MINOCA, and 104 patients (48.5%) in stenosis. CHD is more susceptible over the age of 50 due to the development of atherosclerosis and additional risk factors^16^.

The study showed an increase in monocytes and stenosis in the MINOCA group, although not statistically significant. Increased monocytes are associated with the severity of coronary atherosclerosis due to the role of monocytes in inflammation, thrombosis, and endothelial dysfunction^17^. HDL values were low in all groups, although not statistically significant, but HDL had anti-inflammatory, antioxidant, and antithrombotic effects and inhibited monocyte activation and differentiation. The coronary artery diameter was smaller in diabetic patients than in hypertension, with significant differences, especially in LAD, LCx, and RCA. The research showed that CHD patients with diabetes had a smaller coronary diameter and a larger total plaque volume than patients without diabetes, affecting the coronary diameter^18^. The larger diameter of the coronary artery in hypertensive patients is caused by changes in the structure of the coronary wall due to increased blood pressure, which results in coronary turbulent flow^19^.

The Table 2 study showed that the average number of leukocytes and monocytes, relative and absolute, was higher in MINOCA and stenosis patients than in coronary slow flow patients. However, these differences were not statistically significant. These findings are consistent with previous studies showing that an increase in monocytes is associated with the severity of coronary atherosclerosis, as circulating monocytes serve as a significant source of pro-inflammatory and pro-oxidant factors^20^.

Mean levels of total cholesterol, LDL, and triglycerides were also higher in MINOCA and stenosis patients, but there was no significant difference between the three groups. HDL levels were found to be low in all groups of patients. HDL has anti-inflammatory, antioxidant, and antithrombotic effects and inhibits the activation and differentiation of monocytes into macrophages, ultimately limiting the inflammatory response^21^. Overall, no parameter showed a statistically significant difference with a *p*-value of >0.05, suggesting that although there was an increasing trend of inflammatory and lipid parameters in patients with coronary obstruction, the variation between groups was insufficient to achieve statistical significance.

The study Table 3 showed significant differences in coronary artery diameter between three groups of patients: coronary slow flow, MINOCA, and stenosis. These findings suggest that patients with slow coronary flow have wider coronary vessels than patients with MINOCA and stenosis. Previous research has shown that smaller coronary artery diameters are associated with an increased risk of atherosclerosis and cardiovascular events^22^. Deseive (2019) reported that patients with diabetes had smaller coronary artery diameters and greater total plaque volume than patients without diabetes, which was in line with the findings of smaller diameters in the stenosis group in this study^23^. This decrease in artery diameter may be due to the further process of atherosclerosis and higher plaque complexity in patients with stenosis and MINOCA, which can contribute to increased vascular resistance and decreased coronary blood flow^24^. Camici (2015) reported the hypertension has also been reported to affect coronary slow flow, which results in impaired heart function^25^.

This study showed a significant difference in coronary artery diameter between stenosis patients with comorbidities of diabetes mellitus (DM) and hypertension (Table 4). These findings suggest that patients with DM have a higher risk for narrowing of the coronary arteries compared to patients with hypertension. Previous research by Shaheen et al. revealed that CHD patients with diabetes have a smaller coronary artery diameter and a greater total plaque volume compared to patients without diabetes, which suggests that diabetes contributes to the development of more severe atherosclerosis. In addition, research by Simon et al. showed a significant difference in total plaque volume (TPV) between patients with diabetes and those without diabetes, with diabetic patients having a larger TPV, which affects the diameter of the coronary arteries^26^.

The narrowing of coronary artery diameter in patients with diabetes may be due to increased insulin resistance, dyslipidemia, and a more potent inflammatory mechanism compared to hypertension^27^. Meanwhile, hypertension can also cause structural changes to the walls of the coronary arteries that increase the risk of atherosclerosis^28^. Still, the effects may not be as strong as in patients with diabetes^29^. The results of this study confirm that diabetes is a more substantial risk factor for the narrowing of the coronary arteries compared to hypertension, in line with previous findings.

The study found that the Monocyte to HDL Ratio (MHR) values were higher in patients with coronary slow flow, MINOCA, and stenosis compared to the normal group, as shown in the dot-plot graph (Figure 3). Increased MHR is associated with atherosclerosis lesions’ presence, progression, severity, and slow flow in the coronary arteries^30^. These findings are consistent with previous studies that show a relationship between high MHR and the severity of coronary artery lesions in patients with acute coronary syndrome (SKA) and chronic inflammation in atherosclerosis and slow coronary flow (SCF)^31^. High MHR is also a significant predictor of chronic total occlusion (CTO) incidence in CHD patients, signaling high inflammatory and oxidative processes and decreased protective effects on atherogenic^32^.

High MHR in the stenosis group indicates significant monocyte activation and lower anti-inflammatory effects of HDL. Monocyte activation is important in initiating atherosclerosis, where the number of monocytes in circulation can predict the development of new plaques^33^. Monocyte accumulation at plaque sites is related to increased expression of adhesion molecules such as VCAM-1. Platelets and neutrophils also help recruit monocytes to plaques^34^. HDL has antiatherosclerotic effects that inhibit monocyte activation and anti-inflammatory, antioxidant, and antithrombotic effects. HDL also suppresses monocyte activity, inhibits monocyte differentiation into macrophages, limits the inflammatory response, and contains the enzyme paraoxonase 1, which protects against LDL oxidation in the blood vessel walls^35^.

Table 5 shows that the relationship between MHR and coronary artery diameter in the coronary slow flow group showed a sufficient correlation in distal LAD and proximal and distal RCA. However, it was statistically insignificant with a *p*-value >0.05. Another study found a significant difference between a decreased RML value and a change in coronary artery diameter in the CSF and NCSF groups^36^. In addition, an increase in MHR and a decrease in RML were independent parameters in the slow flow/no-reflow group in patients with non-ST myocardial elevation infarction (NSTEMI)^37^. In the MINOCA group, no significant relationship was found between MHR and coronary artery diameter. In the stenosis group, the relationship between MHR and Left Main diameter, proximal and distal LAD, and distal RCA showed a weak and statistically insignificant correlation with a *p*-value >0.05.

The absence of a significant association between MHR and coronary artery diameter in these three groups is likely due to the high prevalence of hypertension: 76% in the coronary slow flow group, 33.3% in the MINOCA group, and 41% in the stenosis group. Hypertension can cause vascular remodeling associated with changes in lumen diameter, either decrease or increase, which are classified as internal and external remodeling^38^. Vascular remodeling that increases the diameter of the lumen can affect the median diameter figure, so the relationship between the variables becomes insignificant when tested statistically^39^. It suggests that although there is a relationship between MHR and some coronary artery parameters, hypertension, and vascular remodeling may obscure those relationships in statistical analysis.

This study highlights the Monocyte-HDL Ratio (MHR) as an important marker of systemic inflammation and lipid imbalance in cardiovascular disease (CVD). Elevated monocyte counts and reduced HDL levels in stenosis patients demonstrate the role of inflammation and lipid dysregulation in atherosclerosis progression through foam cell formation and impaired cholesterol transport. Stenosis patients with diabetes mellitus (DM) showed smaller coronary artery diameters compared to those with hypertension, indicating the influence of DM in accelerating atherosclerosis through oxidative stress and endothelial dysfunction, while hypertension-induced vascular remodeling contributed to changes in coronary dimensions. Although no significant correlation between MHR and coronary artery diameter was observed, elevated MHR in stenosis and MINOCA patients highlights its potential as a marker of atherosclerotic burden and a predictor of adverse cardiovascular events, particularly in high-risk groups like DM and hypertension patients. Further research is needed to confirm these findings and explore its clinical applications.

## Conclusions

This study concluded that the monocyte-HDL ratio in coronary heart disease (CHD) patients in the coronary slow flow, MINOCA, and stenosis groups was higher compared to the normal group. However, no significant relationship was found between MHR and coronary artery diameter in the three groups. Specifically, the analysis showed no significant correlation between MHR and coronary artery diameter in the stenosis, coronary slow flow, or MINOCA groups. These results indicate that although high MHR is associated with the presence and progression of atherosclerosis, the effect of MHR on coronary artery diameter is not significant enough to be a single diagnostic parameter in this context.

## Limitations

This study’s limitations include an imbalanced sample size, a very small MINOCA group, and a single-center setting, which limit generalizability. The cross-sectional design prevents causal inference, and reliance on medical records may affect data accuracy. Other factors like vascular remodeling and plaque characteristics were not thoroughly evaluated. Future research with larger, balanced, and multicenter samples is needed to validate these findings.

## AUTHOR STATEMENTS

Conception: Azhari Gani, Muhammad Diah Yusuf, Malahayati.

Design: Azhari Gani, Muhammad Diah Yusuf, Siti Adewiyah

Supervision: Azhari Gani, Muhammad Diah Yusuf

Funding: Azhari Gani, Malayahati

Materials: Azhari Gani, Muhammad Diah Yusuf, Siti Adewiyah, Malahayati

Data Collection and/or Processing: Azhari Gani, Malahayati

Analysis and/or Interpretation: Azhari Gani, Siti Adewiyah, Malahayati

Literature Review: Azhari Gani, Malahayati

Writing: Azhari Gani, Malahayati

Critical Review: Azhari Gani, Muhammad Diah Yusuf, Siti Adewiyah, Malahayati

## COMPETING INTEREST

The authors declare there are no competing interests.

## Acknowledgement

The Authors thanks the RSU ZA Hospital and the students of the Internal Medicine Specialist Doctor Education Program of Universitas Syiah Kuala for all their knowledge and support in this process and all the participants who have supported and provided information for this study.
